# The Tissue-Selective Estrogen Complex: A Review of Current Evidence

**DOI:** 10.1007/s40744-015-0013-z

**Published:** 2015-05-20

**Authors:** Rinu Pazhekattu, Arthur N. Lau, Jonathan D. Adachi

**Affiliations:** 1 PGY1 Internal Medicine, McMaster University, Hamilton, ON Canada; 2 Division of Rheumatology, Department of Medicine, McMaster University, Hamilton, ON Canada

**Keywords:** Bazedoxifene, Bone mineral density, Conjugated estrogens, Osteoporosis, Menopause, Tissue-selective estrogen complex

## Abstract

The tissue-selective estrogen complex (TSEC) has recently entered the market for the treatment of postmenopausal osteoporosis, and is particularly targeted to women with significant vasomotor symptoms. This review appraises the evidence behind the only approved TSEC to-date, a combination of bazedoxifene and conjugated estrogens, with regards to its efficacy and relevant safety concerns. The majority of evidence that has led to its approval is derived from the SMART study. This large phase III trial with several substudies was aimed at discerning the effects of the TSEC on various estrogen-responsive tissues in comparison to raloxifene and placebo. Overall, the evidence thus far suggests a superior improvement in lumbar bone mineral density of 1.01% ± 0.28% as well as decrease in the frequency of hot flushes. Regarding safety concerns, endometrial thickness did not change over the treatment course, and investigators also identified a modest reduction in breast density. While there was no difference in rates of venous thromboembolism between treatment and placebo groups in a 2-year follow-up period, the effects of the drug on coagulation profiles are similar to those seen with hormone replacement therapy. Thus, the drug’s effects on venous thromboembolism risk over a longer treatment course remain unclear. In conclusion, the actual efficacy of the TSEC for postmenopausal osteoporosis remains as yet undefined, given the lack of fracture prevention data. The evidence thus far does seem to suggest a beneficial effect on vasomotor symptoms and a generally favorable side effect profile. However, it should be noted that only one study has addressed this question thus far, and so the repeatability of the findings is still in question.

**Electronic supplementary material:**

The online version of this article (doi:10.1007/s40744-015-0013-z) contains supplementary material, which is available to authorized users.

## Osteoporosis: The Burden of Illness

Osteoporosis is perhaps one of the most under-recognized chronic diseases when considering its prevalence and its associated morbidity and mortality. In Canada, osteoporotic fractures are more common than the combined incidence of heart attacks, strokes, and breast cancer, representing a very large disease burden [1, 2]. An estimated one-third of women and one-fifth of men will incur an osteoporotic fracture in their lifetime, and the disease represents 3.9 billion dollars in cost to the Canadian health care system [1, 2]. The skeletal affliction is highly prevalent internationally as well. In Europe and the United States, there are 1.7 million and 0.3 million hip fractures per year, respectively, with the overwhelming majority of these representing osteoporotic fractures [2]. Not only are these fractures highly prevalent, but patients who sustain hip or vertebral fractures also have higher mortality rates [3].

## Osteoporosis and Menopause

The risk factors for osteoporosis are long and varied, but postmenopausal women remain the largest cohort of those affected [4]. Both genders begin to lose bone with advancing age, but estrogen deficiency leads to an accelerated rate of bone loss in postmenopausal women. Estrogen acts to promote osteoblast activity. Additionally, it inhibits the maturation of osteoclast precursors and induces osteoclast apoptosis. Through these combined effects estrogen plays a critical role in maintaining bone integrity and normal bone turnover [5]. The postmenopausal state is characterized by a relative deficiency of estrogen and therefore results in accelerated bone loss. Given that postmenopausal women are at greatest risk for osteoporosis, this population has attracted particular interest in research on osteoporosis prevention and treatment.

## Current Therapeutic Guidelines

**Fig. 1:**
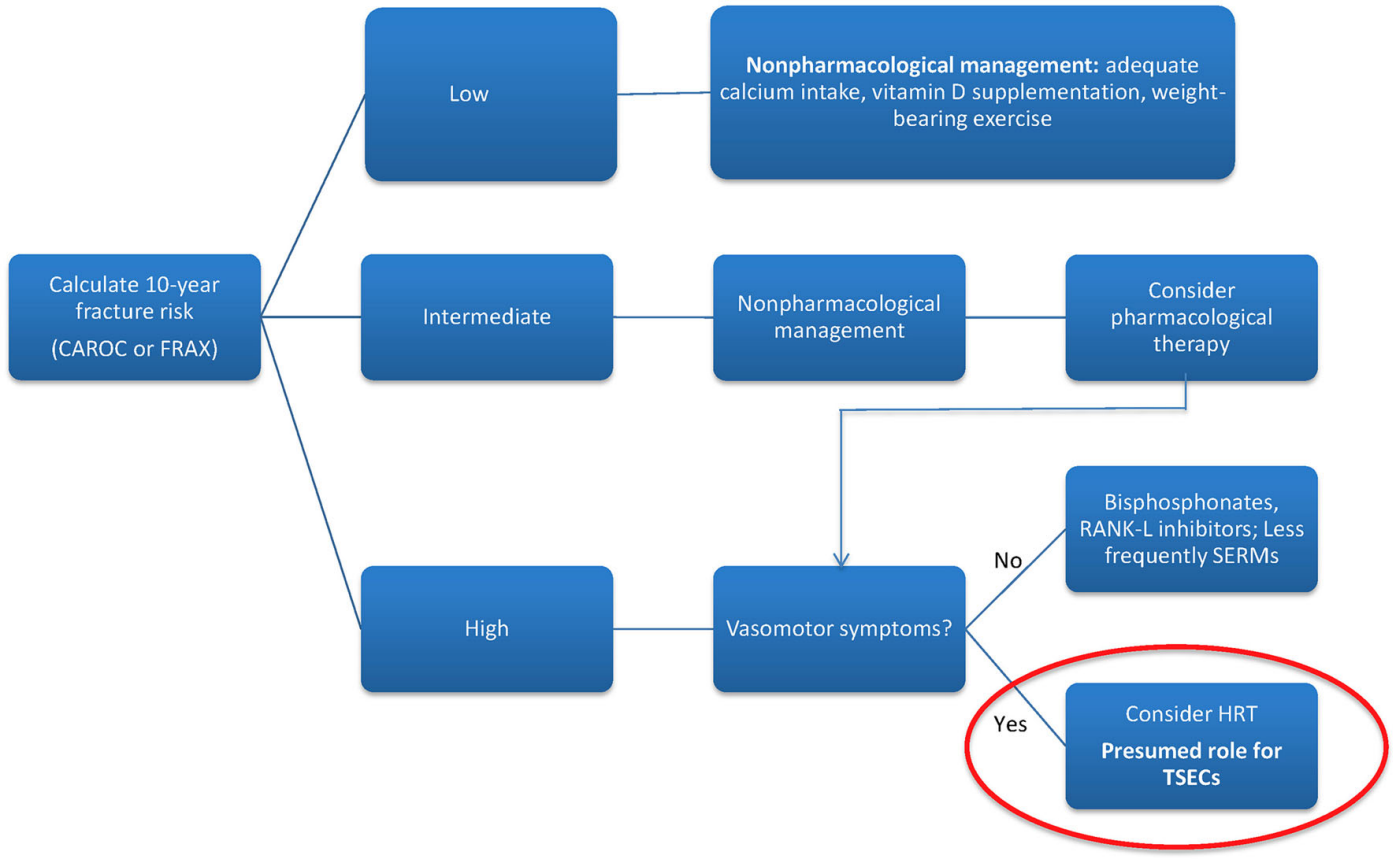
Osteoporosis treatment algorithm. *CAROC* Canadian Association of Radiologists and Osteoporosis Canada, *FRAX* fracture risk assessment tool, *HRT* hormone replacement therapy, *SERM* selective estrogen receptor modulators, *TSEC* tissue-selective estrogen complex

Screening for osteoporosis with a dual X-ray absorptiometry scan to assess bone mineral density (BMD) is recommended for postmenopausal women with significant risk factors, including age over 65 or low body mass index, as well as women over 50 years of age with a preexisting fragility fracture. Clinicians may use the World Health Organization fracture risk assessment tool (FRAX) or another risk assessment framework, like the Canadian Association of Radiologists and Osteoporosis Canada (CAROC) tool to predict 10-year fracture risk [6]. Current treatment regimens for high-risk patients include both lifestyle modifications and pharmacologic options (see Fig. 1). A variety of medications are available, differing in mechanism of action, effectiveness, and side effect profile. These include bisphosphonates, the mainstay of therapy, selective estrogen receptor modulators (SERMs), hormone replacement therapy (HRT), and biologic therapy with RANKL inhibition, according to the most recent Canadian clinical practice guidelines [7, 8]. Bisphosphonates and denosumab, a RANKL inhibitor, are the most frequently prescribed first-line therapies.

Regarding guidelines for prevention, lifestyle modifications, including adequate dietary calcium and vitamin D are routinely recommended, but there is ongoing controversy about whether pharmacologic therapy is indicated in those at moderate risk of fracture [4, 5]. Given the long-term side effects and concerns over attenuation of treatment effectiveness of available therapeutic options, patients in the intermediate risk group present a challenge. Additionally, there is a lack of evidence to guide specialists on preventative treatment. Current guidelines in Canada suggest that pharmacologic agents may be considered in agreeable patients in this intermediate category [4, 6, 9].

## The Role of Hormone Replacement Therapy

HRT once played an integral role in the prevention and treatment of postmenopausal osteoporosis (PMO). Since the Women’s Health Initiative (WHI) study, however, there has been a significant shift in perspective [10]. The side effect profile has greatly altered the risk–benefit balance of HRT. Evidence has repeatedly suggested an increased risk of stroke, thromboembolic events, and especially breast cancer with prolonged use of HRT. Additionally, lower doses of HRT do not produce significant gains in BMD. Accordingly, its use in the management of osteoporosis has declined precipitously.

## Tibolone

Tibolone, a synthetic steroid, was introduced as an option for the management of perimenopausal vasomotor symptoms. Its exact mechanism of action is unclear, but the drug is theorized to elicit its effects by means of variable estrogenic, progestogenic and androgenic activity. At low doses, tibolone has been shown to reduce bone turnover, increase both spine and hip BMD, and reduce spinal fracture risk [11]. A Cochrane review on tibolone’s activity for postmenopausal symptoms suggests lower efficacy compared with HRT in the treatment of postmenopausal symptoms [12]. Safety concerns with HRT were mirrored with tibolone as well, particularly increased risk of stroke in women over 60 years old and increased risk of breast cancer recurrence in patients with previous breast malignancy [12].

## Selective Estrogen Receptor Modulators

Selective estrogen receptor modulators (SERMs) came about as a potential alternative to HRT with a more desirable side effect profile, achieved via the variable action of SERMs on different estrogen receptor-expressing tissues. SERMs differ in their tissue-specific effects. The ideal activity would produce antagonism in the breast and endometrium, mitigating the risk of breast and endometrial cancer seen with HRT. Conversely, they should act as estrogen receptor agonists in the bone and cardiovascular systems, thus maintaining the estrogenic effect on bone health [4]. These differential properties led to approval for the use of certain SERMs in the treatment of PMO.

Raloxifene is the oldest SERM in use for the treatment of PMO. Two large studies, the MORE (http://ClinicalTrials.gov number, NCT00670319) and RUTH (http://ClinicalTrials.gov number, NCT00190593) trials, both showed a reduction in vertebral fracture risk [13]. While there is no primary evidence to support reduction in nonvertebral fractures, a post hoc secondary analysis suggested nonvertebral efficacy in those with the greatest vertebral deformity [14]. The perimenopausal period may also pose a challenge for its use, as evidence suggests increased incidence of hot flashes among women treated with raloxifene [12, 13]. Additionally, there was a reduced risk of breast cancer in postmenopausal women with osteoporosis treated with raloxifene [15]. In terms of effects on the endometrium, the MORE trial did reveal an increased incidence of endometrial polyps with raloxifene use; however, multiple studies suggest that this does not translate to an increased the risk of endometrial hyperplasia or malignancy [16, 17]. Finally, the increased risk of venous thromboembolism (VTE) compared to placebo is still a concern [18].

Bazedoxifene (BZA) is a newer generation SERM indicated in the treatment of PMO. A large, long-term, multicenter trial showed BZA to be effective in reducing vertebral fractures [19]. While there was also an improvement in both lumbar and total hip bone mineral densities, the drug failed to produce a tangible reduction in nonvertebral fractures [19]. The effects of the drug were particularly significant in women with higher fracture risk. In fact, a subgroup analysis suggested possible benefit even for nonvertebral fractures in this subset of women [20]. Thus, BZA has established itself as a viable alternative to raloxifene in the treatment of PMO.

Additionally, a recent network meta-analysis showed that BZA provides comparable risk reduction to bisphosphonates for nonvertebral fractures in women at higher risk of fracture based on their FRAX score [21]. However, this was an indirect comparison of the two medications, and a randomized-controlled trial directly comparing the two drugs is needed to confirm these results.

Regarding the safety of BZA, studies suggest that the drug is generally tolerable and safe, even with long-term use. As expected, BZA did not increase the incidence of breast, endometrial or ovarian cancers [22]. However, as with other SERMs, the main adverse events (AEs) include a higher incidence of VTEs. Furthermore, while the drug is generally well-tolerated in nonflushing postmenopausal women [23], evidence suggests that BZA significantly exacerbates vasomotor symptoms, particularly in perimenopause [20]. This poses an obvious challenge in terms of tolerability and compliance in postmenopausal women who are affected by hot flushes.

## Development of the Tissue-Selective Estrogen Complex

The tissue-selective estrogen complex, or TSEC, was created in recognition of the need for a drug that can concurrently relieve vasomotor symptoms and treat PMO, while avoiding the negative impacts of HRT alone. The TSEC blends conjugated estrogens (CE) with a SERM, aiming to retain the beneficial effects of estrogen, while the SERM component acts to diminish its harmful effects on the breast and endometrium [24]. The experimental goal was to achieve a combination that worked synergistically to preserve bone health.

Various SERM/CE combinations had been explored in both in vivo and in vitro studies; however, BZA showed superior competitive inhibition of CE in breast and endometrial tissues, as well as promising effectiveness and safety profile during testing in monkeys [15, 17, 18, 25–27]. The combination of 20 mg BZA and 0.45 mg CE (Duavee^®^; Pfizer, Mission, KS, USA) is the sole approved drug in this class. It entered the market in October 2013, following approval by the Food and Drug Administration [28]. The main evidence leading to its approval was derived from the SMART (http://ClinicalTrials.gov number, NCT00808132) trial, consisting of five large phase III multicenter substudies evaluating the effectiveness and safety profile of BZA/CE. The following sections will expand on the current evidence behind BZA/CE and its differential effects on various estrogen-responsive tissues.

### Bone Health

The primary indication for the TSEC is purported to be the prevention of PMO. As such, the osteoporosis substudy of the SMART-1 trial focused specifically on bone health and relevant endpoints [29]. This 2-year international multicenter randomized double-blind placebo and active-controlled phase III trial involved a total of 2315 patients spread across two substudies: the first evaluated subjects who were >5 years post-menopause while the second enrolled those 1–5 years post-menopause. There were eight subgroups in each substudy, involving six different combined doses of BZA/CE as well as raloxifene and placebo. Subjects required a baseline BMD score between −1.0 and −2.5 as well as one additional risk factor for osteoporosis to meet inclusion criteria. The trial’s primary endpoint was change in lumbar spine BMD at 2 years, with change in total hip BMD and measured serum bone turnover markers (BTM) as secondary endpoints. The investigators found a significant increase in BMD versus placebo for all BZA/CE doses; however, higher doses of CE provided a more significant change, while increasing doses of BZA tended to dampen the effect. For the current approved dose, 20 mg BZA/0.45 mg CE, there was an adjusted annual increase in lumbar spine BMD of 1.01% ± 0.28%, which was significantly greater than both placebo and raloxifene, with responder rates remaining higher at 24 months versus raloxifene. In reference to secondary endpoints, all doses of BZA/CE produced improvement in total hip and femoral neck BMD versus placebo; however, there was no significant difference from raloxifene for the approved formulation [29]. Of interest, the SMART-5 trial (*N* = 1843) compared BZA/CE to combined estrogen–progestin therapy and found that BMD improvements were greater in the latter group [30]. Finally, there was a decrease in BTM for the relevant dose of BZA/CE when compared to both placebo and raloxifene, suggesting reduced osteoclast activity [29].

### Menopausal Symptoms

Both the SMART-1 and SMART-2 trials assessed the effect of BZA/CE on vasomotor symptoms and analyzed other parameters, including quality of life and sleep. The SMART-1 trial enrolled 3397 subjects divided into the eight previously mentioned subgroups, and showed a significant reduction in both daily hot flash frequency and severity that persisted into the second year of therapy [31, 32]. While there were no significant differences in frequency of sexual activity between treatment and controls, rates of dyspareunia were reduced among those in treatment groups. Finally, all intervention groups were noted to have improvements in vaginal atrophy compared with both controls [31].

These results were confirmed in the SMART-3 trial (*N* = 664), which focused on subjects with vaginal and vulvar atrophy [33, 34]. The SMART-2 trial (*N* = 332), although smaller and shorter, revealed similar outcomes. Significant reductions in hot flush frequency and severity were evident by week 4 for both doses of BZA/CE tested, and by week 12, there was a 74% reduction in daily hot flashes for the approved BZA/CE dose. Notably, responder rates were quite high for achievement of 50% reduction in hot flush frequency [35, 36]. Outcomes related to sleep, assessed by the Medical Outcomes Study (MOS) sleep scale, and quality of life, assessed by the menopause-specific quality of life (MENQOL) questionnaire, were also favorable [37, 28, 38].

### Endometrial Proliferation

The risk of endometrial cancer associated with unopposed estrogen therapy is prohibitive to its use, even in patients with significant vasomotor symptoms. Thus, the effect of TSECs on endometrial proliferation are of key interest and likely to affect prescribing practice. The SMART-1 trial sought to answer this question, and found that incidence of endometrial hyperplasia was <1% and that no significant change in endometrial thickness was appreciated from baseline compared to 2 years with 20 mg BZA/0.45 mg CE. Notably, at least 20 mg BZA was required to prevent endometrial hyperplasia [29, 30]. The SMART-5 trial, which included comparison with combined estrogen–progestin therapy, did not confirm these results entirely. While rates of hyperplasia were still measured to be <1%, there was a significant increase from baseline thickness of 0.17 mm as well as a significant increase in endometrial polyps; however, atypia was absent in all specimens, and there were no cases of endometrial carcinoma. Furthermore, the rates of all endometrial endpoints were similar between BZA/CE and combined estrogen–progestin therapy [30].

Investigators also observed rates of uterine bleeding and amenorrhea, in consideration of pertinent side effects of HRT that prompt treatment termination. Amenorrhea rates (>93% by cycles 10–13) were similar between treatment and placebo groups over the 2-year period [39]. This suggests that BZA/CE may have better tolerability, which has implications for patient compliance.

## Breast Density Changes

Increased risk of breast cancer remains a chief concern when considering HRT. The TSEC is proposed to provide a protective effect in this regard. Thus, an ancillary, retrospective study gathered mammographic evidence of SMART-1 participants to determine breast density changes after treatment with BZA/CE. Investigators found similar baseline breast densities among all treatment arms, and a modest but significant decrease of less than 0.5% was identified at 24 months with 20 mg BZA/0.45 mg CE [40]. This certainly supports the conjecture that the TSEC not only mitigates the risk of breast cancer, but may also counteract it.

### Cardiovascular Effects

The effects of HRT on the cardiovascular system have yet to be clearly defined. While previous studies have suggested a protective role, the WHI study not only failed to confirm this postulation, but also showed that the reverse may be true. However, critics point out that a great proportion of subjects were older and more distantly postmenopausal, potentially confounding results [41]. Recent studies have shown that estrogens may actually produce favorable changes in lipid profile, thereby altering coronary disease risk factors [42]. SERMs, BZA included, are thought to exert similar effects on lipid profiles [43].

In light of this ongoing controversy, both the SMART-1 and SMART-5 trials sought to evaluate the cardiovascular effects of BZA/CE. The SMART-5 trial found that total cholesterol and low-density lipoprotein (LDL) levels were similarly decreased with both BZA/CE and HRT. Conversely, high-density lipoprotein (HDL) levels were increased in both groups. Interestingly, while there was a significant increase in triglyceride levels in the HRT group, there was no significant difference in BZA/CE at 12 months [21, 36]. The SMART-1 trial confirmed the effects of BZA/CE on total cholesterol, LDL and HDL; however, in contradiction of the SMART-5 trial, there was a significant increase in triglyceride levels at the 2-year mark compared to placebo. Additionally, there was no difference in the incidence of cardiovascular events between treatment and placebo groups at 2 years [31]. Overall, these results tend to suggest advantageous effects on the lipid profile with BZA/CE therapy.

### Coagulation Profile

VTEs are recognized complications of HRT and SERMs [44]. Previous trials have shown an elevated incidence of VTEs with BZA use as well [45]. The SMART trials essentially showed a neutral effect on hemostatic mechanisms. However, changes in coagulation profiles were similar to HRT in the SMART-5 trial [46]. Thus, it is difficult to predict the effect of BZA/CE on VTE rates based solely on its effects on coagulation markers. The SMART-1 trial directly measured rates of VTEs and found no difference between treatment arms and placebo [31]. Still, it should not escape notice that the SMART-1 trial was conducted over 2 years, while a previous phase III trial on BZA alone did show more frequent VTEs over 5 years of therapy. While the results of the SMART-1 trial suggest promising evidence that VTE risk may not present a barrier to use of BZA/CE, further confirmatory evidence is needed before drawing strong conclusions in either direction.

### Side Effects and Adverse Events

Rates of AEs were similar between the study drug and placebo, including no significant differences in rates of ischemic stroke, VTE, and coronary heart disease at study termination [31]. Only six deaths occurred, and were presumed to have no connection to the study drug [28]. The causes of these deaths were bronchoaspiration, intracerebral hemorrhage, metastatic lung cancer, chronic obstructive pulmonary disease, and two cases of accidental death [31]. The most common AEs requiring treatment included headache, infection, pain, and arthralgia, and there was no difference in their incidence between treatment groups and placebo. Furthermore, in the majority of cases, these AEs were thought to be unrelated to the study drug [35]. Combined with the neutral effect on endometrial tissue and potentially protective breast density changes, these results suggest that BZA/CE is a safe therapeutic option.

In terms of tolerability, breast pain, a common side effect of HRT associated with discontinuation of treatment, did not appear at a greater frequency in treatment arms. As mentioned earlier, uterine bleeding appeared with lower frequency in the BZA/CE as opposed to HRT, suggesting a better side effect profile [47, 48].

## Conclusions: Where Will the Tsec Fit in Clinical Practice?

Regarding use of the drug in patients requiring vasomotor symptom relief, the evidence so far is in support of BZA/CE as a safer therapeutic alternative to HRT when breast and endometrial outcomes are considered. Furthermore, it may be better tolerated than hormonal therapy alone, with fewer treatment-limiting side effects. A summary of the overall effects of BZA/CE versus HRT and SERMs is provided in Table 1.

**Table 1:** Estrogen-related effects of the TSEC in comparison to raloxifene and HRT

Pharmacologic intervention	TSEC	Raloxifene	HRT
Bone health	↑ [29]^b^	↑ [18, 50]^a^	↑ [10, 51]^b^
Endometrial hyperplasia	Neutral [49]^b^	Varies [50, 52]^a^	↑ [17]^a^
Breast cancer risk	Unclear [40]^b^	↓ [15, 53]^b^	↑ [4, 10]^a^
Vasomotor symptoms	↓ [31, 35]^b^	↑ [18]^b^	↓ [54]^a^
Stroke risk	Unclear [31]^c^	Neutral [18, 50, 55]^b^	↑ [4, 10]^a^
VTE risk	Unclear [31]^b^	↑ [18, 50]^a^	↑ [4, 10]^a^

*HRT* hormone replacement therapy, *SERM* selective estrogen receptor modulators, *TSEC* tissue-selective estrogen complex, *VTE* venous thromboembolism

^a^Grade 1A evidence

^b^Grade 2A evidence

^c^Grade 2C evidence

↑ denotes an increase in the relevant outcome

↓ denotes a decrease in the relevant outcome

In reference to bone health outcomes, current evidence is relatively encouraging. The improvement in BMD with the approved formulation of BZA/CE is, at the very least, comparable to raloxifene. As these trials were centered on evaluating efficacy with a preventative goal, the gain in BMD alone suggests it may be effective in delaying progression to a high-risk fracture group. However, more research is needed that considers patient-important outcomes related to osteoporosis, namely fracture prevention data. Such data would help to clarify the role of BZA/CE not only in the prevention, but also in the treatment of osteoporosis. The role of BZA/CE in the treatment algorithm for PMO as compared to the other existing treatment options still remains unclear.

The novelty of the medication means that long-term data on safety are not yet available. Perhaps, like HRT, the risk profile becomes increasingly concerning with longer use and older age. In this case it may be a great alternative to HRT for vasomotor symptoms in early postmenopausal women, but may not provide lasting bone health improvements. Without these outcomes, it is difficult to predict what role BZA/CE will play in osteoporosis prevention, especially when considering the controversy among experts on whether pharmacologic prevention is indicated. In postmenopausal women with bothersome menopause-related symptoms, BZA/CE appears to be a better alternative to HRT, and may provide an additional bone-protective benefit. In the absence of vasomotor symptoms, there may be a role for BZA/CE in postmenopausal women at moderate risk for fracture in the 60–70 years age range, during which time vertebral fractures are more prevalent; however, after the age of 70 years, hip fractures become a greater concern and the drug’s clinical utility with respect to hip fractures needs further clarification.

Finally, the evidence surrounding the efficacy and safety of BZA/CE is based entirely on preclinical studies and a single phase III trial assessing multiple outcomes. Before any strong conclusions can be drawn, the reproducibility of these effects must be determined. Therefore, while the current evidence suggests a possible role for BZA/CE in the treatment algorithm for osteoporosis, further evaluation is required to confirm or refute the findings in this single study.

This review article was based on previously conducted studies and does not involve any new studies of human or animal subjects performed by any of the authors.

## Electronic supplementary material

Below is the link to the electronic supplementary material.

Supplementary material 1 (PDF 186 kb)
