# Supplementary material for: The Tissue-Selective Estrogen Complex: A Review of Current Evidence
Source: Rheumatol Ther. 2015 May 20;2(1):47–58. doi: 10.1007/s40744-015-0013-z (PMC4883253; doi:10.1007/s40744-015-0013-z)
Supplement: Supplementary file 1 — Supplementary material 1 (PDF 186 kb) [file 40744_2015_13_MOESM1_ESM.pdf]

- The tissue selective estrogen complex (TSEC) is a novel class of medications, which are aimed at preventing and treating postmenopausal osteoporosis and peri-menopausal vasomotor symptoms without the harmful effects of HRT.
- Data regarding the safety & efficacy of the only approved TSEC to-date, 20 mg bazedoxifene (BZA)/0.45 mg conjugated estrogens (CE), are derived from the SMART trials, five large interrelated phase III studies.
- The safety profile, particularly the cardiovascular, endometrial, and hemostatic effects, tends to be reassuring but long-term data is not yet available.
- The effects on bone are promising given the bone mineral density improvements, but no fracture prevention data are currently available.
- BZA/CE may be a reasonable choice for treatment of vasomotor symptoms in early postmenopausal patients that may provide additional benefit in a subset of osteoporotic patients at risk for vertebral fractures.
- As all these results are drawn from a single study and further trials confirming these results are required before firm recommendations can be made.

This summary slide represents the opinions of the authors. No funding or sponsorship was received for this study or publication of this article. For a full list of acknowledgments and conflicts of interest for all authors of this article, please see the full text online. Copyright © The Author(s) 2015. Creative Commons Attribution Noncommercial License (CC BY-NC).
